# (2-Acetyl­phenolato)(2-{1-[2-(morpholin-4-yl)ethyl­imino]­eth­yl}phenolato)(thio­cyanato-κ*N*)­cobalt(III)

**DOI:** 10.1107/S1600536811025116

**Published:** 2011-06-30

**Authors:** Chen-Yi Wang

**Affiliations:** a Department of Chemistry, Huzhou University, Huzhou 313000, People’s Republic of China

## Abstract

The title mononuclear cobalt(III) complex, [Co(C_14_H_19_N_2_O_2_)(C_8_H_7_O_2_)(NCS)], was obtained by the reaction of 2-acetyl­phenol, 2-(morpholin-4-yl)ethyl­amine, ammonium thio­cyan­ate and cobalt nitrate in methanol. The Co^III^ atom is coordinated by one phenolate O, one imine N, and one amine N atom of the tridentate Schiff base ligand, two O atoms of the 2-acetyl­phenolate anion and one thio­cyanate N atom. This results in a fairly regular *fac*-CoN_3_O_3_ octa­hedral coordination geometry for the metal ion. The dihedral angle between the two benzene rings is 88.3 (3)°.

## Related literature

For background to urease inhibitors, see: Wang (2009 ▶); Wang & Ye (2011 ▶). For similar cobalt(III) complexes, see: Li *et al.* (2007 ▶, 2008 ▶); Liu (2010 ▶); Wu *et al.* (2011 ▶).
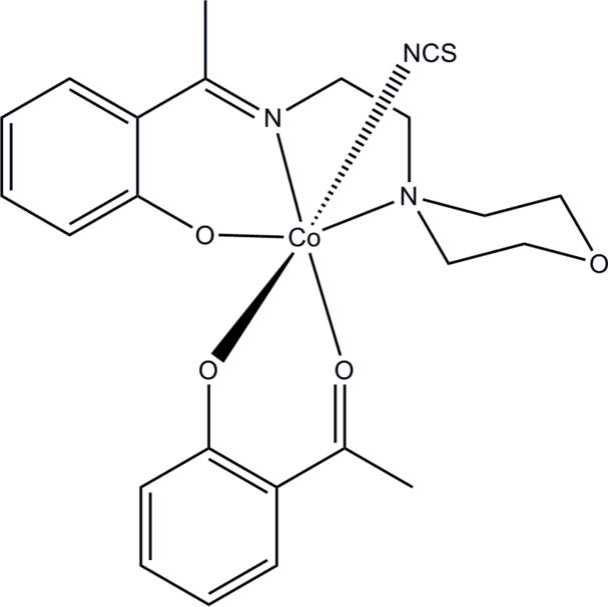


## Experimental

### 

#### Crystal data

[Co(C_14_H_19_N_2_O_2_)(C_8_H_7_O_2_)(NCS)]
*M* *_r_* = 499.46
Monoclinic, 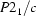

*a* = 8.145 (2) Å
*b* = 15.801 (2) Å
*c* = 17.702 (3) Å
β = 102.687 (3)°
*V* = 2222.6 (7) Å^3^
*Z* = 4
Mo *K*α radiation
μ = 0.90 mm^−1^
*T* = 298 K
0.32 × 0.30 × 0.28 mm

#### Data collection

Bruker SMART CCD diffractometer
Absorption correction: multi-scan (*SADABS*; Sheldrick, 1996 ▶) *T*_min_ = 0.761, *T*_max_ = 0.786
13159 measured reflections
4588 independent reflections
2764 reflections with *I* > 2σ(*I*)
*R*_int_ = 0.047

#### Refinement

*R*[*F* ^2^ > 2σ(*F* ^2^)] = 0.041
*wR*(*F* ^2^) = 0.101
*S* = 1.03
4588 reflections
291 parameters
H-atom parameters constrained
Δρ_max_ = 0.24 e Å^−3^
Δρ_min_ = −0.22 e Å^−3^

### 

Data collection: *SMART* (Bruker, 1998 ▶); cell refinement: *SAINT* (Bruker, 1998 ▶); data reduction: *SAINT*; program(s) used to solve structure: *SHELXS97* (Sheldrick, 2008 ▶); program(s) used to refine structure: *SHELXL97* (Sheldrick, 2008 ▶); molecular graphics: *SHELXTL* (Sheldrick, 2008 ▶); software used to prepare material for publication: *SHELXTL*.

## Supplementary Material

Crystal structure: contains datablock(s) global, I. DOI: 10.1107/S1600536811025116/hb5932sup1.cif

Structure factors: contains datablock(s) I. DOI: 10.1107/S1600536811025116/hb5932Isup2.hkl

Additional supplementary materials:  crystallographic information; 3D view; checkCIF report

## Figures and Tables

**Table 1:** Selected bond lengths (Å)

Co1—O1	1.9094 (19)
Co1—O2	1.8621 (18)
Co1—O3	1.8624 (19)
Co1—N1	1.894 (2)
Co1—N3	1.914 (3)
Co1—N2	2.054 (2)

**Fig. 1.**
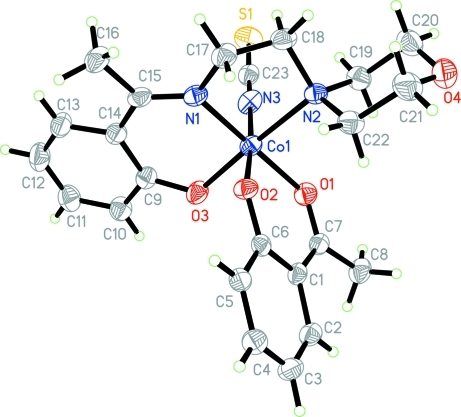
The molecular structure of the title compound, showing displacement ellipsoids drawn at the 30% probability level.

## supplementary crystallographic information

### Comment

As part of our investigations into urease inhibitors (Wang & Ye, 2011; Wang, 2009), we have synthesized the title compound, a new mononuclear cobalt(III) complex, Fig. 1. The Co atom in the complex is six-coordinated by one phenolate O, one imine N, and one amine N atoms of a Schiff base ligand 2-[1-(2-morpholin-4-ylethylimino)ethyl]phenolate, two O atoms of 2-acetylphenolate, and one thiocyanate N atom, forming an octahedral geometry. The dihedral angle between the two benzene rings are 88.3 (3) °. The three *trans* angles at Co atom are in the range 175.1 (1)–177.5 (1)°; the other angles are close to 90°, ranging from 84.4 (1) to 94.2 (1)° (Table 1), indicating a slightly distorted octahedral coordination. The Co–O and Co–N bond lengths (Table 1) are typical and are comparable with those observed in other similar cobalt(III) complexes (Li *et al.*, 2007; Liu, 2010; Li *et al.*, 2008; Wu *et al.*, 2011).

### Experimental

2-Acetylphenol (1.0 mmol, 0.136 g), 2-morpholin-4-ylethylamine (0.5 mmol, 0.065 g), ammonium thiocyanate (1.0 mmol, 0.076 g), and cobalt nitrate hexahydrate (0.5 mmol, 0.145 g) were dissolved in MeOH (30 ml). The mixture was stirred at room temperature for 10 min to give a clear deep brown solution. After keeping the solution in air for a week, brown block-shaped crystals were formed at the bottom of the vessel.

### Refinement

All H atoms were placed in geometrically idealized positions and constrained to ride on their parent atoms, with C—H distances in the range 0.93–0.97 Å, and with *U*_iso_(H) set at 1.2*U*_eq_(C) and 1.5*U*_eq_(methyl C).

### Crystal data

**Table:**

[Co(C_14_H_19_N_2_O_2_)(C_8_H_7_O_2_)(NCS)]	*F*(000) = 1040
*M**_r_* = 499.46	*D*_x_ = 1.493 Mg m^−^^3^
Monoclinic, *P*2_1_/*c*	Mo *K*α radiation, λ = 0.71073 Å
*a* = 8.145 (2) Å	Cell parameters from 2215 reflections
*b* = 15.801 (2) Å	θ = 2.5–24.5°
*c* = 17.702 (3) Å	µ = 0.90 mm^−^^1^
β = 102.687 (3)°	*T* = 298 K
*V* = 2222.6 (7) Å^3^	Block, brown
*Z* = 4	0.32 × 0.30 × 0.28 mm

### Data collection

**Table:**

Bruker SMART CCD diffractometer	4588 independent reflections
Radiation source: fine-focus sealed tube	2764 reflections with *I* > 2σ(*I*)
graphite	*R*_int_ = 0.047
ω scans	θ_max_ = 26.5°, θ_min_ = 2.6°
Absorption correction: multi-scan (*SADABS*; Sheldrick, 1996)	*h* = −10→9
*T*_min_ = 0.761, *T*_max_ = 0.786	*k* = −15→19
13159 measured reflections	*l* = −21→22

### Refinement

**Table:**

Refinement on *F*^2^	Primary atom site location: structure-invariant direct methods
Least-squares matrix: full	Secondary atom site location: difference Fourier map
*R*[*F*^2^ > 2σ(*F*^2^)] = 0.041	Hydrogen site location: inferred from neighbouring sites
*wR*(*F*^2^) = 0.101	H-atom parameters constrained
*S* = 1.03	*w* = 1/[σ^2^(*F*_o_^2^) + (0.0387*P*)^2^ + 0.1364*P*] where *P* = (*F*_o_^2^ + 2*F*_c_^2^)/3
4588 reflections	(Δ/σ)_max_ < 0.001
291 parameters	Δρ_max_ = 0.24 e Å^−^^3^
0 restraints	Δρ_min_ = −0.22 e Å^−^^3^

### Special details

**Table:**

Geometry. All e.s.d.'s (except the e.s.d. in the dihedral angle between two l.s. planes) are estimated using the full covariance matrix. The cell e.s.d.'s are taken into account individually in the estimation of e.s.d.'s in distances, angles and torsion angles; correlations between e.s.d.'s in cell parameters are only used when they are defined by crystal symmetry. An approximate (isotropic) treatment of cell e.s.d.'s is used for estimating e.s.d.'s involving l.s. planes.
Refinement. Refinement of *F*^2^ against ALL reflections. The weighted *R*-factor *wR* and goodness of fit *S* are based on *F*^2^, conventional *R*-factors *R* are based on *F*, with *F* set to zero for negative *F*^2^. The threshold expression of *F*^2^ > σ(*F*^2^) is used only for calculating *R*-factors(gt) *etc*. and is not relevant to the choice of reflections for refinement. *R*-factors based on *F*^2^ are statistically about twice as large as those based on *F*, and *R*- factors based on ALL data will be even larger.

### Fractional atomic coordinates and isotropic or equivalent isotropic displacement parameters (Å^2^)

**Table:**

	*x*	*y*	*z*	*U*_iso_*/*U*_eq_	
Co1	0.10480 (5)	0.75033 (2)	0.40903 (2)	0.04240 (14)	
N1	0.2071 (3)	0.84863 (14)	0.45991 (14)	0.0451 (6)	
N2	0.2519 (3)	0.76544 (13)	0.32944 (13)	0.0423 (6)	
N3	−0.0691 (3)	0.81713 (16)	0.34625 (15)	0.0553 (7)	
O1	0.0119 (2)	0.64890 (12)	0.35843 (11)	0.0511 (5)	
O2	0.2783 (2)	0.69333 (11)	0.47566 (10)	0.0445 (5)	
O3	−0.0305 (2)	0.73184 (12)	0.47967 (12)	0.0545 (6)	
O4	0.3888 (3)	0.69744 (15)	0.20174 (14)	0.0762 (7)	
S1	−0.30242 (11)	0.92290 (6)	0.25423 (5)	0.0708 (3)	
C1	0.1654 (3)	0.55174 (17)	0.45072 (16)	0.0406 (7)	
C2	0.1808 (4)	0.46685 (18)	0.47754 (18)	0.0481 (7)	
H2	0.1094	0.4261	0.4501	0.058*	
C3	0.2958 (4)	0.44293 (19)	0.54166 (18)	0.0542 (8)	
H3	0.3012	0.3870	0.5585	0.065*	
C4	0.4056 (4)	0.5034 (2)	0.58189 (17)	0.0510 (8)	
H4	0.4868	0.4873	0.6251	0.061*	
C5	0.3955 (3)	0.58572 (18)	0.55864 (16)	0.0432 (7)	
H5	0.4701	0.6249	0.5866	0.052*	
C6	0.2756 (3)	0.61332 (18)	0.49350 (16)	0.0399 (7)	
C7	0.0426 (3)	0.57306 (18)	0.38210 (17)	0.0429 (7)	
C8	−0.0548 (4)	0.50563 (19)	0.33162 (17)	0.0603 (9)	
H8A	−0.1453	0.5311	0.2947	0.090*	
H8B	−0.0997	0.4662	0.3631	0.090*	
H8C	0.0185	0.4763	0.3047	0.090*	
C9	−0.0742 (4)	0.79083 (19)	0.52384 (16)	0.0465 (7)	
C10	−0.2153 (4)	0.7738 (2)	0.55482 (18)	0.0576 (9)	
H10	−0.2744	0.7236	0.5419	0.069*	
C11	−0.2673 (4)	0.8297 (2)	0.60370 (19)	0.0673 (9)	
H11	−0.3606	0.8169	0.6239	0.081*	
C12	−0.1830 (5)	0.9046 (2)	0.62323 (19)	0.0711 (10)	
H12	−0.2208	0.9431	0.6553	0.085*	
C13	−0.0427 (4)	0.9222 (2)	0.59514 (17)	0.0614 (9)	
H13	0.0156	0.9722	0.6100	0.074*	
C14	0.0158 (4)	0.86696 (18)	0.54447 (16)	0.0468 (7)	
C15	0.1688 (4)	0.88750 (17)	0.51812 (17)	0.0473 (7)	
C16	0.2859 (4)	0.95472 (19)	0.56150 (17)	0.0659 (9)	
H16A	0.3996	0.9422	0.5584	0.099*	
H16B	0.2779	0.9553	0.6148	0.099*	
H16C	0.2544	1.0091	0.5388	0.099*	
C17	0.3581 (4)	0.86927 (19)	0.43098 (18)	0.0557 (8)	
H17A	0.4518	0.8344	0.4566	0.067*	
H17B	0.3883	0.9282	0.4414	0.067*	
C18	0.3200 (4)	0.85313 (18)	0.34579 (17)	0.0535 (8)	
H18A	0.4217	0.8595	0.3263	0.064*	
H18B	0.2381	0.8940	0.3196	0.064*	
C19	0.1570 (4)	0.75906 (19)	0.24703 (16)	0.0548 (8)	
H19A	0.0748	0.8043	0.2363	0.066*	
H19B	0.0967	0.7057	0.2397	0.066*	
C20	0.2705 (5)	0.7645 (2)	0.19053 (18)	0.0676 (10)	
H20A	0.2031	0.7622	0.1381	0.081*	
H20B	0.3298	0.8181	0.1971	0.081*	
C21	0.4903 (4)	0.7038 (2)	0.2774 (2)	0.0716 (10)	
H21A	0.5525	0.7566	0.2820	0.086*	
H21B	0.5712	0.6578	0.2857	0.086*	
C22	0.3886 (3)	0.70057 (19)	0.33915 (17)	0.0545 (8)	
H22A	0.3387	0.6448	0.3388	0.065*	
H22B	0.4638	0.7085	0.3893	0.065*	
C23	−0.1659 (4)	0.86167 (19)	0.30900 (18)	0.0475 (8)	

### Atomic displacement parameters (Å^2^)

**Table:**

	*U*^11^	*U*^22^	*U*^33^	*U*^12^	*U*^13^	*U*^23^
Co1	0.0373 (2)	0.0384 (2)	0.0506 (3)	−0.00521 (18)	0.00765 (17)	−0.00324 (19)
N1	0.0408 (14)	0.0395 (14)	0.0524 (16)	−0.0071 (11)	0.0048 (12)	−0.0005 (12)
N2	0.0401 (13)	0.0395 (15)	0.0455 (14)	−0.0035 (11)	0.0054 (11)	0.0020 (11)
N3	0.0447 (16)	0.0499 (16)	0.0682 (19)	0.0013 (13)	0.0055 (14)	−0.0069 (14)
O1	0.0479 (12)	0.0509 (14)	0.0541 (13)	−0.0085 (10)	0.0101 (10)	−0.0053 (10)
O2	0.0446 (11)	0.0380 (12)	0.0486 (12)	−0.0066 (9)	0.0053 (9)	0.0015 (10)
O3	0.0544 (13)	0.0459 (13)	0.0688 (15)	−0.0115 (10)	0.0257 (12)	−0.0131 (10)
O4	0.0883 (18)	0.0750 (18)	0.0742 (17)	−0.0075 (15)	0.0372 (15)	−0.0125 (14)
S1	0.0555 (5)	0.0781 (7)	0.0741 (6)	0.0045 (5)	0.0040 (5)	0.0192 (5)
C1	0.0392 (16)	0.0378 (17)	0.0484 (18)	−0.0029 (13)	0.0175 (14)	−0.0080 (14)
C2	0.0455 (17)	0.0415 (18)	0.061 (2)	−0.0057 (15)	0.0198 (16)	−0.0056 (16)
C3	0.061 (2)	0.0387 (18)	0.066 (2)	0.0023 (16)	0.0217 (18)	0.0027 (16)
C4	0.0480 (18)	0.053 (2)	0.0512 (19)	0.0055 (16)	0.0098 (15)	0.0027 (16)
C5	0.0437 (17)	0.0459 (18)	0.0406 (17)	−0.0035 (14)	0.0104 (14)	−0.0018 (14)
C6	0.0390 (16)	0.0432 (18)	0.0419 (18)	−0.0041 (14)	0.0187 (14)	−0.0045 (14)
C7	0.0395 (16)	0.0409 (18)	0.0525 (19)	−0.0070 (14)	0.0192 (14)	−0.0069 (15)
C8	0.061 (2)	0.056 (2)	0.063 (2)	−0.0134 (17)	0.0116 (17)	−0.0185 (17)
C9	0.0444 (17)	0.0449 (18)	0.0476 (18)	0.0045 (15)	0.0048 (14)	−0.0029 (15)
C10	0.052 (2)	0.061 (2)	0.062 (2)	−0.0026 (16)	0.0190 (17)	−0.0055 (17)
C11	0.066 (2)	0.079 (3)	0.061 (2)	0.008 (2)	0.0220 (19)	−0.003 (2)
C12	0.082 (3)	0.072 (3)	0.061 (2)	0.017 (2)	0.020 (2)	−0.014 (2)
C13	0.079 (2)	0.050 (2)	0.053 (2)	0.0022 (18)	0.0078 (19)	−0.0107 (16)
C14	0.0533 (18)	0.0416 (18)	0.0418 (18)	0.0037 (15)	0.0024 (15)	0.0011 (14)
C15	0.0552 (19)	0.0342 (16)	0.0464 (19)	0.0005 (15)	−0.0023 (16)	0.0064 (14)
C16	0.077 (2)	0.052 (2)	0.062 (2)	−0.0169 (18)	0.0025 (19)	−0.0124 (17)
C17	0.0499 (19)	0.0502 (19)	0.067 (2)	−0.0137 (15)	0.0132 (17)	−0.0100 (17)
C18	0.0531 (19)	0.0438 (19)	0.065 (2)	−0.0103 (15)	0.0165 (16)	0.0020 (16)
C19	0.0546 (19)	0.060 (2)	0.0465 (19)	−0.0074 (16)	0.0047 (15)	0.0000 (16)
C20	0.078 (2)	0.077 (3)	0.048 (2)	−0.015 (2)	0.0146 (18)	−0.0008 (18)
C21	0.060 (2)	0.074 (3)	0.088 (3)	0.006 (2)	0.033 (2)	0.010 (2)
C22	0.0481 (18)	0.052 (2)	0.068 (2)	0.0062 (16)	0.0220 (16)	0.0108 (17)
C23	0.0411 (18)	0.0464 (19)	0.057 (2)	−0.0080 (15)	0.0137 (15)	−0.0085 (16)

### Geometric parameters (Å, °)

**Table:**

Co1—O1	1.9094 (19)	C8—H8C	0.9600
Co1—O2	1.8621 (18)	C9—C10	1.404 (4)
Co1—O3	1.8624 (19)	C9—C14	1.414 (4)
Co1—N1	1.894 (2)	C10—C11	1.368 (4)
Co1—N3	1.914 (3)	C10—H10	0.9300
Co1—N2	2.054 (2)	C11—C12	1.374 (4)
N1—C15	1.295 (3)	C11—H11	0.9300
N1—C17	1.469 (3)	C12—C13	1.371 (4)
N2—C22	1.495 (3)	C12—H12	0.9300
N2—C19	1.497 (3)	C13—C14	1.408 (4)
N2—C18	1.497 (3)	C13—H13	0.9300
N3—C23	1.150 (3)	C14—C15	1.460 (4)
O1—C7	1.276 (3)	C15—C16	1.519 (4)
O2—C6	1.304 (3)	C16—H16A	0.9600
O3—C9	1.315 (3)	C16—H16B	0.9600
O4—C21	1.415 (4)	C16—H16C	0.9600
O4—C20	1.416 (4)	C17—C18	1.493 (4)
S1—C23	1.625 (3)	C17—H17A	0.9700
C1—C2	1.419 (4)	C17—H17B	0.9700
C1—C6	1.424 (4)	C18—H18A	0.9700
C1—C7	1.434 (4)	C18—H18B	0.9700
C2—C3	1.357 (4)	C19—C20	1.506 (4)
C2—H2	0.9300	C19—H19A	0.9700
C3—C4	1.393 (4)	C19—H19B	0.9700
C3—H3	0.9300	C20—H20A	0.9700
C4—C5	1.362 (4)	C20—H20B	0.9700
C4—H4	0.9300	C21—C22	1.510 (4)
C5—C6	1.407 (4)	C21—H21A	0.9700
C5—H5	0.9300	C21—H21B	0.9700
C7—C8	1.500 (4)	C22—H22A	0.9700
C8—H8A	0.9600	C22—H22B	0.9700
C8—H8B	0.9600		
			
O2—Co1—O3	88.70 (8)	C11—C10—H10	119.4
O2—Co1—N1	84.38 (9)	C9—C10—H10	119.4
O3—Co1—N1	94.17 (9)	C10—C11—C12	120.6 (3)
O2—Co1—O1	93.36 (8)	C10—C11—H11	119.7
O3—Co1—O1	87.21 (8)	C12—C11—H11	119.7
N1—Co1—O1	177.31 (9)	C13—C12—C11	119.6 (3)
O2—Co1—N3	175.10 (9)	C13—C12—H12	120.2
O3—Co1—N3	90.27 (10)	C11—C12—H12	120.2
N1—Co1—N3	90.93 (10)	C12—C13—C14	122.1 (3)
O1—Co1—N3	91.38 (9)	C12—C13—H13	119.0
O2—Co1—N2	90.86 (8)	C14—C13—H13	119.0
O3—Co1—N2	177.51 (8)	C13—C14—C9	117.6 (3)
N1—Co1—N2	88.22 (9)	C13—C14—C15	119.9 (3)
O1—Co1—N2	90.37 (8)	C9—C14—C15	122.5 (3)
N3—Co1—N2	90.37 (10)	N1—C15—C14	121.4 (3)
C15—N1—C17	122.6 (2)	N1—C15—C16	120.0 (3)
C15—N1—Co1	127.5 (2)	C14—C15—C16	118.7 (3)
C17—N1—Co1	109.32 (18)	C15—C16—H16A	109.5
C22—N2—C19	106.1 (2)	C15—C16—H16B	109.5
C22—N2—C18	112.2 (2)	H16A—C16—H16B	109.5
C19—N2—C18	109.9 (2)	C15—C16—H16C	109.5
C22—N2—Co1	111.92 (16)	H16A—C16—H16C	109.5
C19—N2—Co1	114.01 (17)	H16B—C16—H16C	109.5
C18—N2—Co1	102.84 (16)	N1—C17—C18	108.0 (2)
C23—N3—Co1	175.3 (2)	N1—C17—H17A	110.1
C7—O1—Co1	127.26 (19)	C18—C17—H17A	110.1
C6—O2—Co1	124.85 (17)	N1—C17—H17B	110.1
C9—O3—Co1	124.42 (18)	C18—C17—H17B	110.1
C21—O4—C20	108.5 (3)	H17A—C17—H17B	108.4
C2—C1—C6	118.0 (3)	C17—C18—N2	109.8 (2)
C2—C1—C7	119.9 (3)	C17—C18—H18A	109.7
C6—C1—C7	122.1 (3)	N2—C18—H18A	109.7
C3—C2—C1	122.5 (3)	C17—C18—H18B	109.7
C3—C2—H2	118.8	N2—C18—H18B	109.7
C1—C2—H2	118.8	H18A—C18—H18B	108.2
C2—C3—C4	119.0 (3)	N2—C19—C20	112.5 (2)
C2—C3—H3	120.5	N2—C19—H19A	109.1
C4—C3—H3	120.5	C20—C19—H19A	109.1
C5—C4—C3	120.8 (3)	N2—C19—H19B	109.1
C5—C4—H4	119.6	C20—C19—H19B	109.1
C3—C4—H4	119.6	H19A—C19—H19B	107.8
C4—C5—C6	121.9 (3)	O4—C20—C19	111.3 (3)
C4—C5—H5	119.0	O4—C20—H20A	109.4
C6—C5—H5	119.0	C19—C20—H20A	109.4
O2—C6—C5	117.0 (3)	O4—C20—H20B	109.4
O2—C6—C1	125.2 (3)	C19—C20—H20B	109.4
C5—C6—C1	117.9 (3)	H20A—C20—H20B	108.0
O1—C7—C1	123.3 (3)	O4—C21—C22	112.6 (3)
O1—C7—C8	115.6 (3)	O4—C21—H21A	109.1
C1—C7—C8	121.1 (3)	C22—C21—H21A	109.1
C7—C8—H8A	109.5	O4—C21—H21B	109.1
C7—C8—H8B	109.5	C22—C21—H21B	109.1
H8A—C8—H8B	109.5	H21A—C21—H21B	107.8
C7—C8—H8C	109.5	N2—C22—C21	114.0 (2)
H8A—C8—H8C	109.5	N2—C22—H22A	108.8
H8B—C8—H8C	109.5	C21—C22—H22A	108.8
O3—C9—C10	116.6 (3)	N2—C22—H22B	108.8
O3—C9—C14	124.3 (3)	C21—C22—H22B	108.8
C10—C9—C14	119.0 (3)	H22A—C22—H22B	107.6
C11—C10—C9	121.1 (3)	N3—C23—S1	178.3 (3)
